# Advantages of Bistable Microwires in Digital Signal Processing

**DOI:** 10.3390/s24082423

**Published:** 2024-04-10

**Authors:** Patrik Jacko, Peter Duranka, Rastislav Varga

**Affiliations:** 1 RVmagnetics a.s., Nemcovej 30, 04001 Košice, Slovakia; patrik.jacko.2@tuke.sk (P.J.); peter.duranka@tuke.sk (P.D.); 2 Faculty of Electrical Engineering and Informatics, Technical University of Košice, 04200 Košice, Slovakia; 3 Centre of Progressive Materials, Technology and Innovation Park, P.J. Safarik University, 04001 Košice, Slovakia

**Keywords:** bistable microwire, magnetic bistability, DSP, digital signal processing, STM32, signal processing

## Abstract

The advantageous applications of magnetic bistable microwires have emerged during long-lasting research. They have a wide range of applications in the scientific sphere or technical practice. They can be used for various applications, including magnetic memories, biomedicine, and sensors. This manuscript is focused on the last-mentioned application of microwires—sensors—discussing various digital signal processing techniques used in practical applications. Thanks to the highly sensitive properties of microwires and their two stable states of magnetization, it is possible to perform precise measurements with less demanding digital processing. The manuscript presents four practical signal-processing methods of microwire response using three different experiments. These experiments are focused on detecting the signal in a simple environment without an external magnetic background, measuring with the external background of a ferromagnetic core, and measuring in harsh conditions with a strong magnetic background. The experiments aim to propose the best method under various conditions, emphasizing the quality and signal processing speed of the microwire signal.

## 1. Introduction

Sensors are one of the key factors for the success of today’s industry. Sensors are traditionally simple devices that convert physical variables into electrical signals or changes in electrical properties. Although this function is a necessary starting point, sensors must also support additional features to function as an integral part of everyday life [1]. They should evolve into something more sophisticated to perform a technically and economically viable role in various industries, including industrial automation, robotics, and precise measurement. The implementation of sensors brings many additional benefits related to predictive maintenance, flexible manufacturing, and improved productivity [1,2,3]. Additionally, they must be cost-effective for deployment on a large scale. Another necessity is for them to be physically small and seamlessly blend into any environment. They should easily handle contactless sensing because wired connections are usually impractical. A further challenging task is very low power consumption so that they can survive for months without battery replacement or harness energy from their surroundings. Moreover, modern sensors should be environmentally resilient, supporting multifunctionality. In the optimum conditions, they also handle self-diagnosis, self-calibration, or the ability to accept calibration commands through wireless connections [3,4,5].

**Figure 1:**
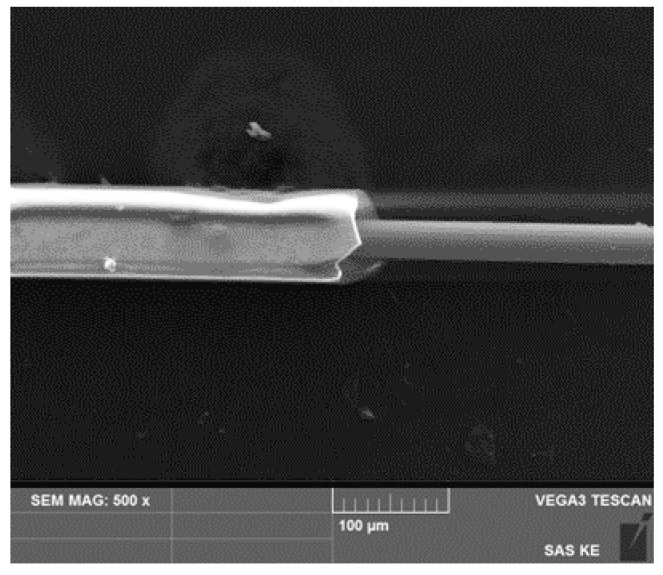
Photograph of a micro wire with the core exposed. Captured using scanning electron microscope at a magnification of 500×.

The requirements for sensors described in the previous paragraph are fulfilled in glass-coated microwires [6,7]. They are composite materials (Figure 1) with a metallic nucleus (diameter from 0.1 to 50 µm) covered by the Pyrex^®^-like glass-coating (thickness 2–20 µm). Magnetic microwires offer advantages such as high sensitivity, low hysteresis, and potential compact construction. They could find applications in various industries, including industrial automation, robotics, and precise measurement [8,9,10]. Additional advantages of microwires are associated with their low weight, miniature dimensions, and low production costs, making them suitable for various aspects of our lives. [9,10] Magnetic microwires with low negative magnetostriction exhibit very weak circular anisotropy [11,12,13,14,15,16]. This is important for the Giant Magneto Impedance effect (GMI), which is a significant change in the impedance of a magnetic material after a change in the applied magnetic field. GMI in magnetic microwires is successfully utilized in magnetic field sensors, motion, and acceleration sensors, electric current sensors, the magnetic encoding of people and goods, and even installed in mobile phones [17,18,19]. These sensors have up to 10 times higher sensitivity than GMR-based sensors, and their dimensions are substantially smaller (micrometer scale) [19,20,21]. From a theoretical perspective, the GMI phenomenon is interesting in studying the properties of magnetic materials using impedance spectroscopy [20,21,22,23].

**Figure 2:**
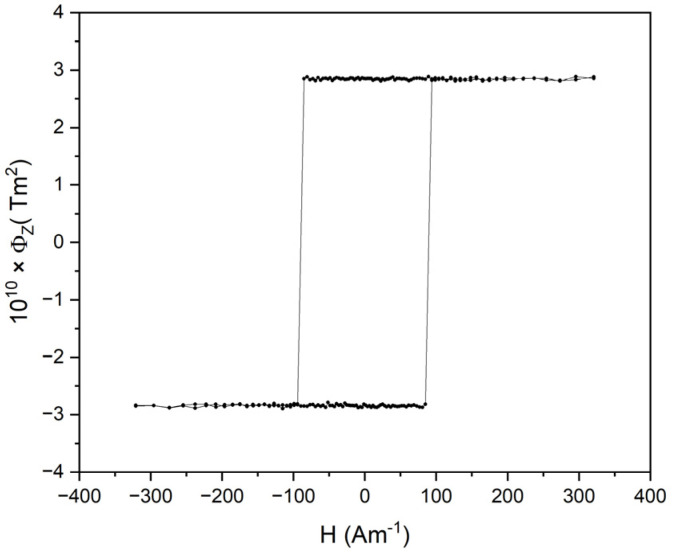
Hysteresis loop of a bistable amorphous microwire with a high positive magnetostriction constant where the *x*-axis presents the magnetic field and *y*-axis presents inductive flux.

Separated paragraphs should be dedicated to the positive magnetostrictive microwires. Due to the (mainly axial) stresses introduced during production, their domain structure consists of a single axially magnetized domain, which is magnetized in the single large Barkhaussen jump of the closure domains that appears naturally to decrease the stray fields [24,25,26,27,28,29,30,31]. As a result, such wires show magnetic bistability, i.e., their magnetization state is characterized by only two states—positive or negative saturation (see Figure 2). The switching between these two states appears at the so-called switching field that corresponds to the depinning field of the closure domain.

The switching field is mainly dependent on the temperature, mechanical stress, or magnetic field. Such dependencies can be used for sensing applications [24,25,26,27,28]. Contrary to the classical magnetic sensors based on permeability variation, bistable wires are characterized by low permeability (comparable to the permeability of a vacuum) [29,30,31,32]. On the other hand, the contribution of the magnetic field, temperature, or stress to the switching field can be easily separated using the triangular shape of the excitation field and properly selected chemical composition. In this case, the bistable wires transfer the measured parameter into the switching time, which is easily digitalized [8]. Generating a periodic signal that can be adjusted by properly selecting the frequency of the excitation field, it is possible to filter the signal even in the case of extremely noisy environments.

Within this manuscript, we demonstrate the advantages of bistable microwires in digital signal processing through four practical methods in three different experiments. They range from sensing in a well-defined environment without an external magnetic background through the signal that needs to be filtered from the magnetic background up to a case where the signal of the microwire needs to be obtained from the harsh conditions of a strong magnetic background. The manuscript compares different processing methods from original data processing through simple averaging and uses an FIR filter and analog comparator. The results compare several microwire signal processing methods regarding accuracy and speed. Based on the advantages found from the individual methods, and provided that the microwire application environment is known, fast and accurate measurements can be performed in real projects.

## 2. Materials and Methods

**Figure 3:**
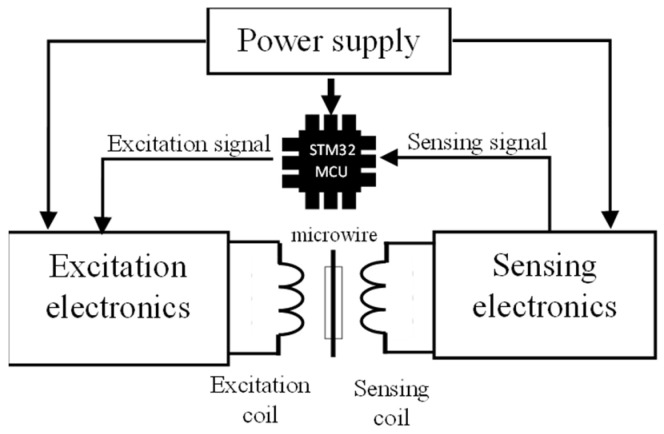
Illustrative block scheme of the microwire measuring system. Microcontroller STM32 is presented as the primary control and processing unit, which generates an excitation signal for excitation electronics. It also processes the sensing signal from the sensing electronics.

The significant advantage of bistable microwires is that, with appropriate circuitry, it is possible to measure multiple physical quantities with high sensitivity. The basis of such a measurement system is a microwire, which is excited by an alternating magnetic field generated by an excitation coil. A sensing coil subsequently captures the signal from the microwire response. The sensing electronics adapt the signal to make it digitally processable by a microcontroller. The microcontroller serves as both an input and output element of the circuit, as it is the source of the signal for generating the magnetic field and also processes the signal. A simple block diagram, with blocks described in the following sections, can be seen in Figure 3.

### 2.1. Magnetic Microwire

The digital processing of microwire signals was conducted using a glass-coated microwire consisting of the chemical composition Fe_76_Si_9_B_10_P_5_. This microwire was prepared using the Taylor–Ulitovsky method [33,34,35,36]. The experiments used a microwire with a length of 30 mm and a core diameter of 14 µm, with a total diameter (including the core and glass coating) of 32 µm (see Figure 1).

### 2.2. Coils System and Switching Time Measurement

The role of the excitation coil is to generate a linearly increasing magnetic field. By increasing the positive or negative magnetic field, a critical field is reached, leading to a change in the domain structure of the microwire. This can be periodically achieved by generating an alternating magnetic field on the excitation coil, which is called the switching field. In the case of the triangular shape of the excitation field, it is directly proportional to the time at which the domain structure changes [37].

**Figure 4:**
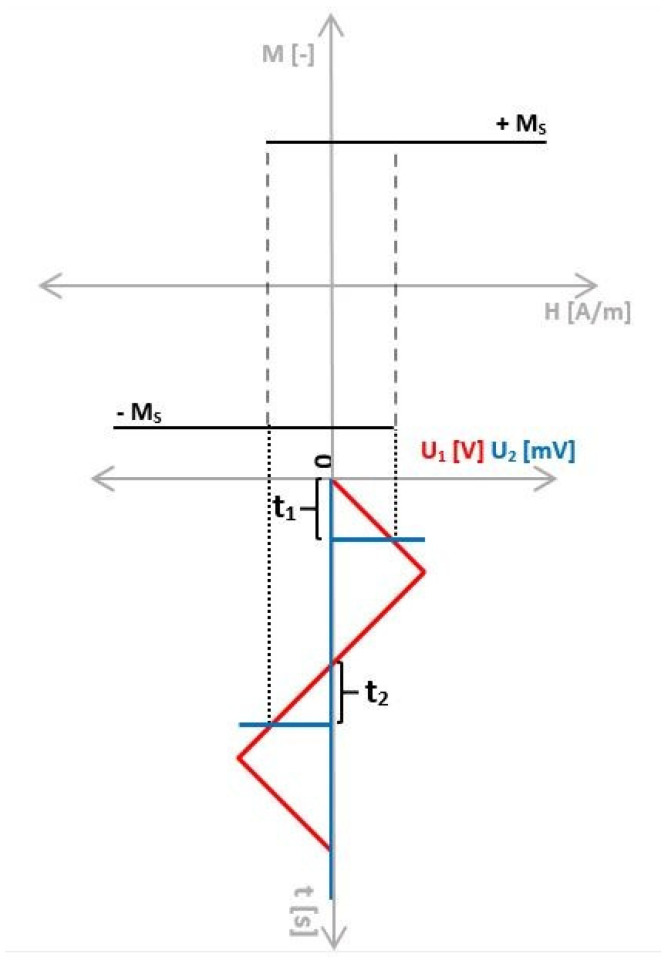
The magnetization process of the bistable microwire and its hysteresis loop. The times *t*_1_ and *t*_2_ represent the times of reaching the critical field (switching field), i.e., switching time [4].

Within the period of the excitation signal, the switching field is represented on the sensing side as an induced microwire response voltage, the position of which can be measured over time. The time intervals measurement presents a fundamental advantage of employing bistable microwires into the measurement chain because the size of the microwire response amplitude is irrelevant, but the time at which the switching field is reached matters. The time at which the switching field is reached is called the switching time (*t*_1_ and *t*_2_ in Figure 4) [24,25,26,27,28,29,30,31].

**Figure 5:**
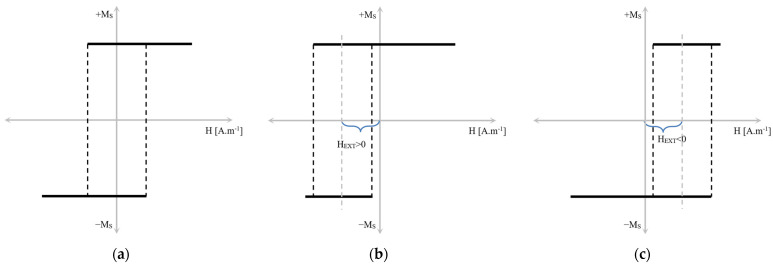
Effect of external magnetic field on bistable microwire hysteresis loop. The loop moves to the left under the influence of a positive magnetic field (**b**) or the right under negative external magnetic field (**c**). The (**a**) part presents hysteresis loop without external field influence [4].

In the scenario where the excitation coil magnetizes the bistable microwire without any additional external magnetic field, *t*_1_ = *t*_2_. Under these conditions, the hysteresis loop is symmetric (as depicted in the above figure). Consequently, the average switching time represents the coercive field, which depends on temperature, pressure, and other parameters [4]. Vice versa, an external magnetic field influence causes the hysteresis loop to shift towards positive or negative polarity depending on the magnitude of the external magnetic field acting upon the microwire (see Figure 5) without any minor hysteresis loops [38]. In this case, the hysteresis loop results in *t*_1_ ≠ *t*_2_. Thanks to this feature, it is possible to measure the magnitude of the external magnetic field by (1), where *H_EXT_* represents H—the external magnetic field.

(1) $$H_{EXT}\;\sim \;\frac{t_{1}-t_{2}}{2}$$

Apart from the external magnetic field’s contribution, other physical quantities derived from the change in the hysteresis loop’s coercivity can be measured. The coercivity of the hysteresis loop is directly influenced by temperature and mechanical stress, which can be measured according to (2), where *H_SW_* presents a switching field.

(2) $$H_{SW}\;\sim \;\frac{t_{1}+t_{2}}{2}$$

It is necessary to mention that temperature or mechanical stress presented as *H_SW_* has a negligible impact on the external magnetic field measurement (*H_EXT_*). On the other hand, the change in the external magnetic field has a negligible impact on the temperature or mechanical stress measurement (*H_SW_*). The influence of *H_EXT_* on the temperature or mechanical stress measurement and vice versa is not absolutely zero. However, it is so small that it can be neglected in the measurement [4,37,39].

The experiment was performed using an induction method based on the excitation (primary) and sensing (secondary) coils, where two types of coil systems were utilized:

- Miniature coil system without a ferromagnetic core.
- Coil system with a ferromagnetic core.

The first coil system—miniature coils—consists of a number of 200 turns, a diameter of 1 mm, and the length of the excitation coil is 10 mm. The parameters of the sensing coil were designed with a number of turns of 150, a diameter of 3 mm, and a length of 1 mm.

**Figure 6:**
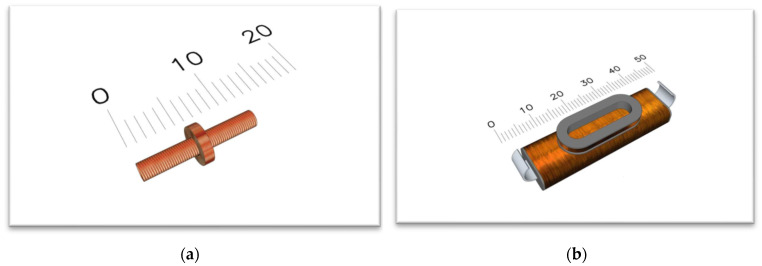
A miniature coil system without a ferromagnetic core (**a**) and a coil system with a ferromagnetic core (**b**) used for generating much larger magnetic fields.

The second coil system consists of a ferromagnetic yoke capable of generating much larger magnetic fields. The parameters of the excitation coil were 6 mm in width and a length of 35 mm with a 1200 number of turns. The sensing coil has a parameter of 1 mm in width, 9 mm in diameter, and a number of 300 turns. Both coil systems are shown in Figure 6.

### 2.3. Microcontroller

It is important to realize that physical quantities directly influencing the coercivity of the hysteresis loop of the microwire, or its shift, are directly proportional to time. This means that measuring the temperature, pressure, or magnetic field does not depend on the microwire response amplitudes but on the time when the domain structure changes. Therefore, to process the switching time accurately, it is necessary to choose a suitable control unit capable of accurately recording this time.

Given these requirements, a high-performance microcontroller STM32G474RE, supporting DSP functions, was chosen for the experiment. The processor core is built on the powerful Arm Cortex-M4, which runs at frequencies of up to 170 MHz. It is the clock source for all peripherals, including essential ones such as comparators, timers, ADC converters, and communication interfaces [40,41].

The microcontroller generates a precise PWM (Pulse-Width Modulation) signal (135 Hz) and serves as the input block of the overall electronic circuitry. An electronics integrator transforms the generated signal into a triangular waveform proportional to the magnitude of the magnetic field applied to the excitation coil. Such a frequency is set due to the slope of the generated magnetic field, which provides the suitable sensitivity of the microwire response.

Simultaneously, the STM32 microcontroller executes the signal processing function induced on the sensing coil. The signal carries information about the precise positions of the measured microwire responses—induced voltage peaks—and, therefore, about the switching time as well. Methods for the processing of the switching time vary depending on factors such as the signal-to-noise ratio, possible interferences, or microwire response signal magnitude. Therefore, several signal-processing methods have been used in experiments where the accuracy, sensitivity, and speed of processing were dominant factors. The signal processing of bistable microwires was, thus, implemented as follows:

- An internal analog comparator processing with a timer of STM32G474RE.
- Single-period processing via an ADC (analog-to-digital converter).
- Sample averaging via ADC.
- The utilization of digital FIR (finite impulse response).

### 2.4. Analog Comparator Processing

The signal processing method through internal analog comparators and the timer of STM32G474RE can be utilized in cases where sufficiently large and distinguishable amplitudes of the microwire responses are present. This technique for processing the switching time is exact, as it relies on the precise timers of the STM32G474RE microcontroller and its accurate integrated analog comparators. The timer records the exact moment when the voltage exceeds the reference value at the internal analog comparator. It occurs due to the change in the magnetization orientation of the microwires, resulting in the mentioned microwire response voltage.

**Figure 7:**
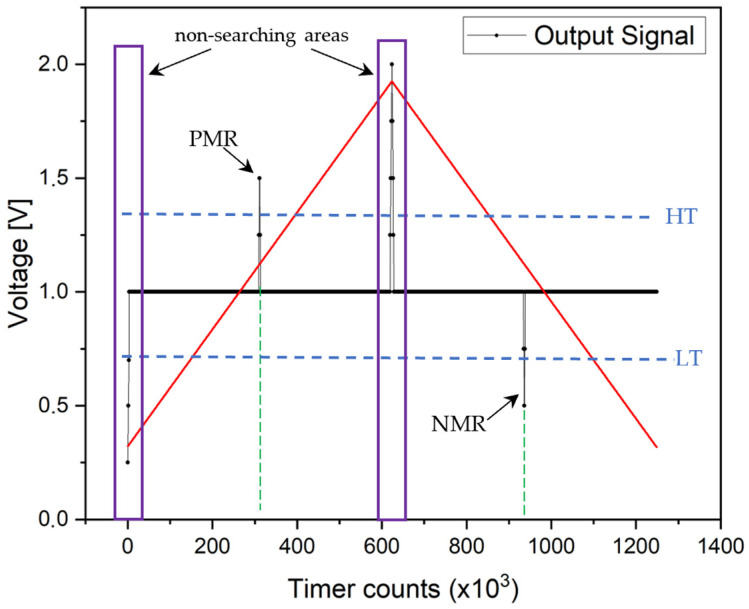
Setting the thresholds to capture the switching time in both positive microwire response (PMR) and negative microwire response (NMR). Areas/times in which the microcontroller does not search for PMR/NMR due to the desired peak from the change in the slope of the excitation signal (red line) are marked as non-searching areas.

The comparator compares the signal voltage with a pair of reference voltages, the so-called high thresholds (HTs) and low thresholds (LTs), as shown in Figure 7. In this case, one comparator was used, which switched the reference voltage to a high or low threshold value after each half-period.

Thanks to the comparator, the microcontroller’s timer can easily record the “switching times” that are shown as a positive microwire response (PMR) and negative microwire response (NMR) in Figure 7. When the comparator detects the achievement of the reference voltage, the microcontroller generates the interruption that stores the timer’s Counter value (CNT) in the memory used. This allows for the accurate measurement of switching times. In the case of an excitation signal (the red triangle wave in Figure 7) with a frequency of 135 Hz and a timer frequency of 170 MHz, a measurement resolution of up to less than 10 ns can be achieved [40,41]. The PWM period is, thus, divided into 1,259,259 measuring points. This means that one half-period within which the microwire response moves is divided in half, that is, into 629,629 measuring points. The times *t*_1_ or *t*_2_ (in µs) can be calculated from the timer counts by (3):

(3) $$t_{1,2}=\frac{CNT}{170}$$

Changing the slope of the triangular signal on the sensing coil creates an unwanted peak at each start and middle of the period. It is not a microwire response but a response to a change in the slope of the excitation signal. Therefore, in every such case, it is necessary to introduce a zone in which no microwire response is sought. These zones are marked as non-searching areas in Figure 7.

### 2.5. Sample Averaging

A unique characteristic of bistable microwires is the periodic generation of the same signal corresponding to the surrounding magnetic field, temperature, or mechanical stress. Thanks to the property of the microwire, it is possible to average signals from multiple periods. It achieves the elimination of unwanted background noise in the signal as well as the possibility of achieving a more accurate result for the switching time.

This relatively simple signal processing method of the bistable microwire is part of the experiment, as it can more precisely define the microwire response (switching time) even in the case of highly noisy signals. In the experiment, the signal averaging of 100 consecutive periods was employed. The entire period of the 135 Hz signal was sampled with 5997 samples, corresponding to a sampling frequency of 810 kHz. The microcontroller unit (MCU) started to sample the signal and store the samples in the memory at the moment of the start of the PWM signaling period. Sampling ended or reset the buffer position at the end of the PWM period, where MCU added a new sample to the previous sample at the given position. Finally, after 100 periods, he divided all samples by the number of periods. The averaging of individual samples was solved according to (4).

(4) $$sample\left(n\right)=\frac{1}{N}\sum_{i=0}^{N}sample(i)$$

In this case, *n* represents the resulting sample after averaging, and *n* = 0,1,2,…, 5996. The number *i* represents the averaging period number (*i* = 1 to 100), and *N* is the total number of averaging—*N* = 100. 

### 2.6. FIR Filtering

The noisy signal on the sensing coil, where the microwave response recognition is complex, can be handled using digital filtering. In these experiments, the signal processing of the microwire was performed using FIR filtering, the main task of which is to remove all useless frequencies or components of a signal. FIR filters help improve signal quality or adapt it to specific application requirements. Digital filtering through an FIR filter results in an output signal consisting of specific and desired frequency components. Thanks to digital filtering, the desired signal can be obtained even from a highly noisy input signal. 

FIR filters are a class of digital filters widely used in signal processing. They characterize a finite-duration impulse response, and their output response to an input signal is determined by a finite number of input samples. 

The operation of FIR filters entails convolving the input signal with a sequence of finite impulse responses, which is represented by a set of coefficients. These coefficients determine the filter’s frequency response characteristics, including its amplitude and phase response. The FIR filters are expressed as Equation (5).

(5) $$y\left(n\right)=\sum_{k=0}^{N-1}b_{k}x(n-k)$$

In this case, *y(n)* represents the output sequence of the filter and *x(n)* represents the inputs of the FIR filter. *N* is the order of the filter, and *b_k_* represents the impulse response value at the *k*-th moment for 0 ≤ *k* ≤ *N* of an *N*-th order filter [42,43,44,45].

The FIR filters offer several advantages, including linear phase response, which ensures that all frequencies in the input signal are delayed equally, making them suitable for applications where phase distortion must be minimized. Additionally, FIR filters can be easily designed to meet specific frequency response requirements, such as low-pass, high-pass, band-pass, or band-stop filtering [46].

**Figure 8:**
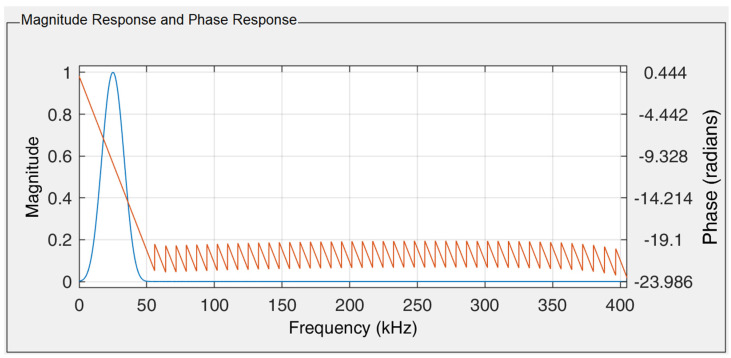
Magnitude response (blue) and phase response (red line) of the designed FIR filter used in the experiment. The FIR filter was designed for a passband from 20 kHz to 30 kHz using the Blackman window.

In this experiment, the purpose of filtering was to remove noise and background low frequencies so that the amplitude of the microwire response was as large as possible. The FIR filter was designed using MATLAB R2023b software and the Filter Designer module. Based on low frequencies and noise (high frequencies) filtering requirements, the band-pass filter was used with 105 FIR coefficients. The filter was designed using the windowing method of filtering through the Blackman window [47,48,49,50]. Since the microwire response frequency is 26 kHz, the filter parameters were set to a band-pass frequency range from 20 kHz to 30 kHz, and the ADC sampling frequency was set to 810 kHz (see Figure 8).

The switching times *t*_1_ and *t*_2_ were calculated according to (6) from methods using samples from the internal ADC,

(6) $$t_{1,2}=\frac{T}{N}sample(n)$$

where *T* represents the period (1/135 Hz), *N* is the total sample number for the period, and *sample* (*n*) represents the sample that resulted in a positive or negative microwire response (*n* = 0,1,2,3, …, 5996). 

## 3. Results

The sensing electronics can influence the signal quality on the sensing side. By employing an analog RC filter circuit, unwanted harmonic components from the output signal can be filtered out. These components may introduce noise or distort the signal background. Analog filters precisely remove these unwanted components, allowing the signal to be processed in the best possible condition. 

However, RC filters have disadvantages, such as the need for additional hardware components on the sensing side and the requirement for redesigning the filter if there are changes in the harmonic components of the background or excitation frequency. On the other hand, with the appropriate filtering of irrelevant background components and noise, highly accurate analog comparators can be used for measurement, thereby enhancing measurement accuracy. 

**Figure 9:**
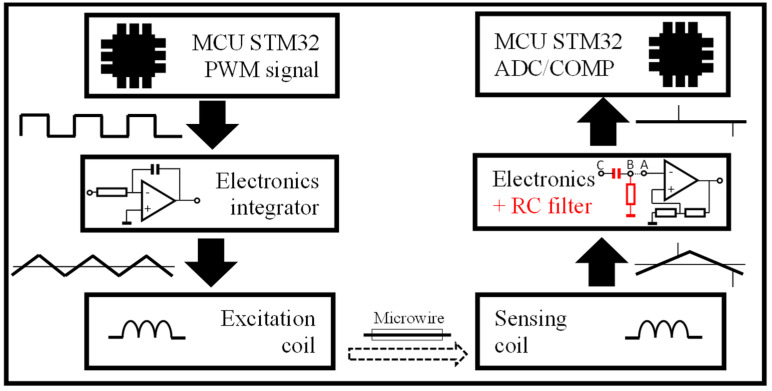
The block scheme of the excitation and sensing parts of a measuring system. When employing the analog RC filter, the signal was routed to point C of the sensing electronics, where A and B points are directly connected. Conversely, when the analog RC filter was not utilized, the signal was directed to point A.

Consequently, all three experiments were conducted with a dual configuration of sensing electronics (see Figure 9—part Electronics + RC filter). In the first configuration, waveforms were measured without an analog RC filter, where the signal bypassed the RC filter and was directly applied to point A. In the second configuration, the signal was applied to the C point, where both A and B points were connected (see Figure 9), resulting in signal filtration.

Thus, a total of three experiments were performed, each containing two electronic configurations (with and without an analog RC filter). Each configuration, in turn, consisted of applying signal processing methods—the evaluation of the microwire response from the original signal, sample averaging, FIR filtering, and, if possible, analog comparators.

### 3.1. Experiment without Magnetic Background

**Figure 10:**
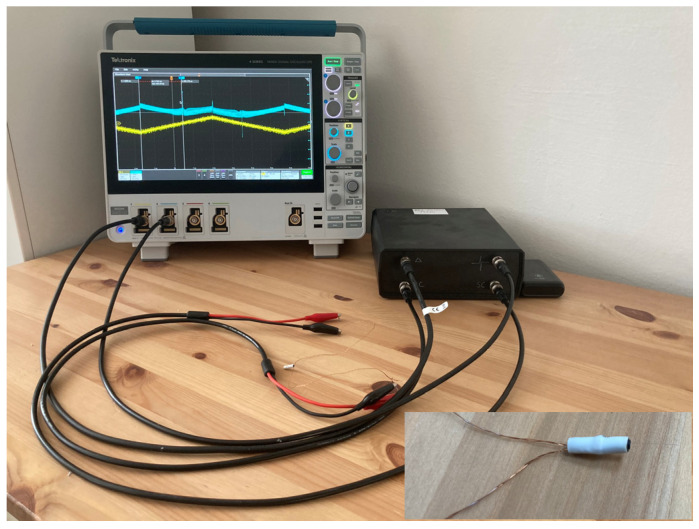
The measurement was conducted using a miniature coil system without a magnetic background. The measurement system schematic includes the black box with the electronics, the coil system with the microwire, and the Tektronix MSO44 oscilloscope, which was used as the microwire responses’ reference position source.

In the simplest measurement scenario, the switching time can be recorded using analog comparators. In this scenario, no external magnetic field or just a small magnetic field is acting on the microwire to distort the signal background. Only a small excitation field is sufficient to excite the microwire, and the amplitudes of the microwire response are large and clearly defined. In the experiment, wooden tables without any metallic parts in the vicinity were used alongside miniature excitation and sensing coils. The arrangement of the pair of coils, as well as the positioning of the microwire in the experimental setup, were implemented according to Figure 10.

In all signal processing methods of the microwire signal by the ADC, predefined regions were specified for microwire response detection. In the first half-period of the processed signal, the STM32 microcontroller searched for the positive microwire response, and in the second half-period, it searched for the negative microwire response. In addition to microwire responses, an additional peak was visible on the signal in all cases, at the beginning and middle of the signal. They arose on the sensing coil as a response to the change in the slope of the triangular signal. Therefore, the microcontroller looked for the sample with the highest and lowest value in the specified area from which the beginning and middle of the period were removed from the search. It ensures that the sample with the higher/smallest value does not change in the slope response of the triangle signal at the beginning or in the middle of the period.

#### 3.1.1. Experiment without Magnetic Background and without an Analog RC Filter

**Figure 11:**
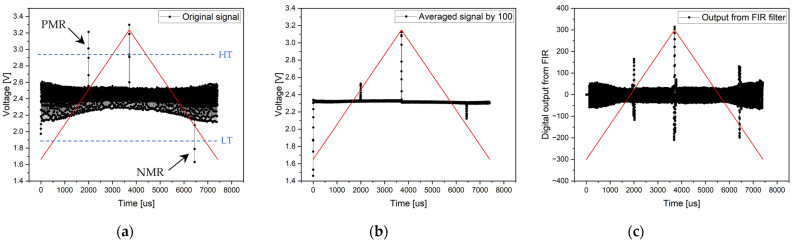
Results of the measurement without a magnetic background and without analog RC filtering. Graph (**a**) shows the original signal of the single-period processing method, the middle chart (**b**) presents the result of an averaged signal from 100 periods, and the right chart (**c**) shows the output from FIR filtering. *HT* represents the high threshold, and *LT* represents the low threshold of the analog comparator.

Electronics without an analog RC filter were used at the beginning of the experimental signal processing from the microwire. Since the signal from the microwire was large and clearly defined, it was possible to use an analog comparator in the experiment to search for microwire response positions. Simultaneously, the signal processing methods via an ADC converter were applied. In the first measurement, the evaluation involved the measurement of a single period of the signal—the original signal. Despite the background noise, the microwire signals were clearly identifiable (see Figure 11. Original signal). As mentioned, the entire period of the 135 Hz signal was sampled with 5997 samples, corresponding to a sampling frequency of 810 kHz. 

In an effort to remove noise and achieve greater measurement accuracy, the second signal processing method of the microwire was employed by averaging the samples. In the experiment, 100 consecutive periods were sampled, and the average value of all samples with the same position was calculated. This resulted in noise suppression and the clear definition of the microwire response. The average signal result can be seen in Figure 11 (with the signal-averaged by 100) and in numerical parameters in Table 1. 

The sample averaging significance is primarily in the microwire’s response distinguishing from the background signal, where noise is suppressed. Naturally, the amplitude may decrease slightly compared to the original signal by averaging samples, digitizing the signal, and minor signal fluctuations from the wire. However, this does not alter the stable position of the microwire’s response over time and ensures a clear differentiation between the microwire response and the background signal.

The final ADC method in this experiment involves signal filtering using an FIR filter. Signal filtering was conducted on a single period with the same sampling frequency as in the previous cases. After filtering, the DC component and all high-frequency and low-frequency signals (noise and background) were removed from the signal except for the microwire response frequencies. However, with an FIR filter, it is necessary to account for the undesired delay of the output signal compared to the input signal caused by the linear phase of the filter. This delay is constant throughout the entire period; thus, it can be compensated for to achieve realistic results of microwire response positions. According to Figure 11 (the output from the FIR filter), the results are shown in Table 1.

Since the microwire responses are very high and clearly defined despite the noise, it is an ideal opportunity to use analog comparators for measurement. During the measurement, these were set to the following threshold values: detecting the positive microwire response at 2870 mV for the negative microwire response at 1900 mV. At these values, 40 consecutive measurements were performed, with the results shown in Table 1. All measurements were compared with a professional Tekronix MSO44 oscilloscope with a bandwidth of 200 MHz and a sample rate of 6.25 GS/s. The cursors were set to measure the peak of the microwire response, and its times are marked as “Reference points.”

#### 3.1.2. Experiment without Magnetic Background and with Analog RC Filter

**Figure 12:**
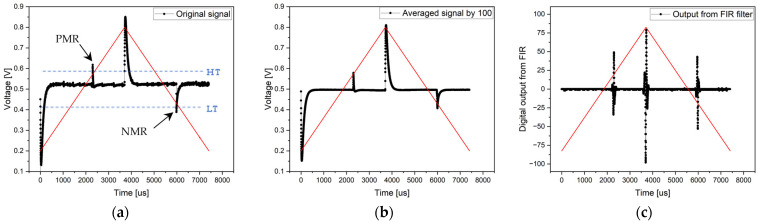
Results of the measurement without a magnetic background with an analog RC filter. Graph (**a**) shows the original signal from the single-period processing method, the middle chart (**b**) presents the result of an averaged signal from 100 periods, and the right chart (**c**) shows the output from FIR filtering.

The same experiment without a magnetic background was conducted using different electronics, including an analog RC filter. Its role was to analogically eliminate all high and low frequencies (the noise and magnetic background). The measurements proceeded in the same steps with the same software configuration of the STM32 microcontroller. The sampling frequency of ADC was 810 kHz, which generated 5997 samples in the 135 Hz period. The results of the experiment conducted with all the above-mentioned signal processing methods are depicted in Figure 12.

The results of the processed signals are immediately clear and distinct. The microwire response is clearly distinguishable even in the case of the original signal (Figure 12. Original signal), without any averaging or applying the FIR filters. The microwire response positions were slightly altered, mainly due to the replacements for other electronics containing RC filters. However, the results remained the same regarding the signal processing quality.

Since the microwire response is sufficiently large, measurements can also be performed using precise analog comparators and a 170 MHz timer. The comparator thresholds were set to a high threshold = 600 mV and a low threshold = 400 mV. The measurement was conducted over 40 consecutive periods. The microcontroller stores the time of the comparator reference voltage (threshold) achievements, resulting in average times from the 40 measurements shown in Table 2. The difference observed in the times compared to the use of ADCs is in the earlier detection of the threshold achievement compared to the observation of the highest point of the microwire response. The threshold achievement occurs before the peak reaches its maximum value. A significant advantage of this measurement method is the exact timing recorded, which is accurate within nanoseconds with a resolution of 5.88 ns [40].

The time efficiency measurement showed that the ideal solution for capturing microwire response is using analog comparators without additional analog RC filters. The results are highly accurate and have a high resolution. Averaging the results from the comparators over multiple periods further stabilizes fluctuations and provides precise measurements. If a visual representation of signals is required, using one of the ADC processing methods without analog RC filters is ideal. For fast measurements, using the single-period processing method is suitable, while for precise measurements, the sample averaging method is more appropriate. The latter stabilizes fluctuations but reduces the amplitude of the microwire responses. Another method providing both speed and accuracy in measurements is FIR filtering, which evaluates the results from a single period, though the microwire response can be influenced by fluctuations. In any case, it is clear from the measurements that additional analog RC filters are not necessary to achieve fast and accurate results.

### 3.2. Experiment with Magnetic Background in Excitation coil

In some cases, it is necessary to generate a large magnetic field. When using a simple coil, generating such a field requires a large excitation current or might even be impossible to achieve. However, generating an equally large magnetic field with a small excitation current is possible by implementing a ferromagnetic core into the excitation coil.

The second experiment focuses on processing the signal of the microwire through a system of coils with a ferromagnetic core capable of generating much larger magnetic fields. As a result, it is possible to excite the microwire from a greater distance or under more demanding conditions. However, the ferromagnetic core directly affects the measurement of the microwire signal because it magnetically acts on the coil system and the microwire, thereby introducing external magnetic fields into the surroundings. This requires compensating for the shift in the excitation magnetic field. Additionally, the ferromagnetic core introduces subtle changes to the background of the captured signal manifested in the sensing coil.

**Figure 13:**
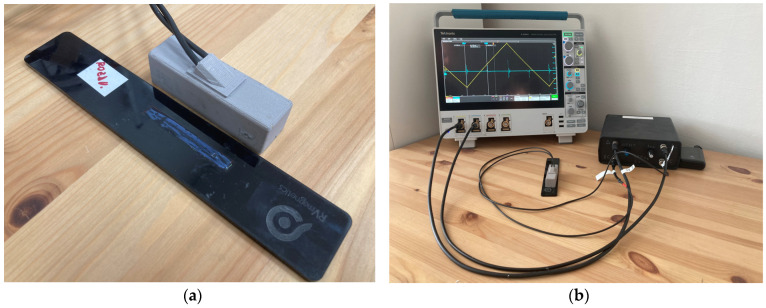
If it is necessary to excite the microwire in an environment requiring a larger magnetic field, it is appropriate to use a coil with transformer laminations. Figure (**a**) depicts the coil system and microwire on a non-magnetic substrate. Figure (**b**) shows the measurement setup with the coil system, microwire, electronics, and Tektronix MSO44 oscilloscope.

In the experiment, the microwire was placed on a non-magnetic (PVC) substrate, on which the system of coils with ferromagnetic cores was also placed. In this case, the only external magnetic field acting on the measured system came from the ferromagnetic core. The arrangement of the measurement system coils and the microwire placed on the PVC substrate can be seen in the following figure (Figure 13).

The experiment was conducted similarly to the previous case in two phases: with and without analog filters. The measurement methods chosen were also identical to the previous experiment as follows: an original signal from the ADC without averaging, the averaging of 100 periods from which the resulting signal was evaluated, and the application of FIR filtering from one period of the signal. If the experiment allowed it, the measurement of switching times was also implemented using the internal analog comparators of the STM32G474RE chip.

#### 3.2.1. Experiment with Magnetic Background in Excitation Coil and without Analog RC Filter

**Figure 14:**
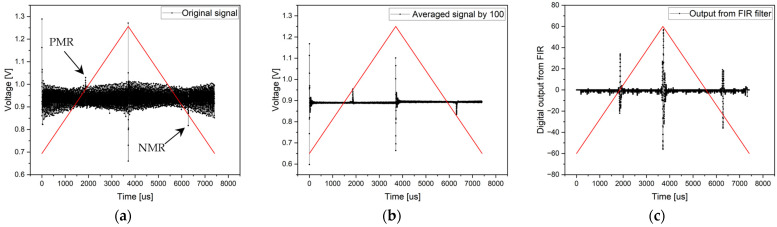
Results of the measurement with a magnetic background in an excitation coil. The electronics are designed without an analog RC filter. Graph (**a**) shows the original signal from the single-period processing method, the middle chart (**b**) presents the result of the averaged signal from 100 periods, and the right chart (**c**) shows the output from FIR filtering.

The ferromagnetic core in the excitation coil caused increased signal noise, making it more challenging to define the microwire response (see Figure 14—original signal). Consequently, analog comparators could not be used to detect the switching time because the microwire response amplitudes were only slightly larger than the background noise. The STM32G474RE microcontroller, based on ADC, evaluated the positions of the microwire response in Table 3. 

Much clearer and better-defined microwire responses were evaluated using the sample averaging method (Figure 14—averaged signal by 100). They are easily distinguishable from the background noise. The time differences (the results compared with the original signal are shown in Table 2) are due to signal digitization, slight fluctuations in the microwire signal, and averaging, as in the previous experiment.

The last signal processing method with coils containing ferromagnetic cores involved signal filtering using an FIR filter. Filtering was applied to a single period of the signal. In this case, the microwire responses were very clearly distinguishable. Their amplitudes were sufficiently large so that the microcontroller could easily determine the microwire responses (see Figure 14—Output from FIR filter). After compensating for the phase shift caused by the FIR filter, the positions of the positive and negative microwire response were determined, as shown in Table 3. 

#### 3.2.2. Experiment with Magnetic Background in Excitation Coil and with Analog RC Filter

**Figure 15:**
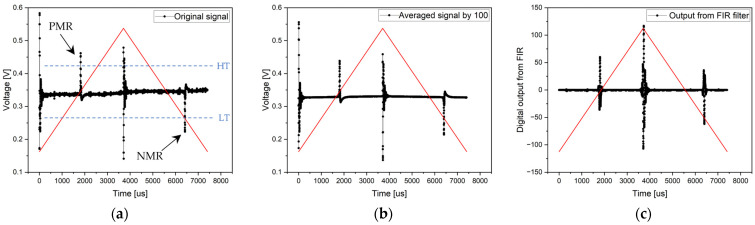
Results of the measurement with a magnetic background in an excitation coil. The electronics contain an analog RC filter. The left graph (**a**) shows the original signal from single-period processing method, the middle chart (**b**) presents the result of the averaged signal from 100 periods, and the right chart (**c**) shows the output from FIR filtering.

Just like in the previous experiment, analog filters also removed high-frequency noise in this case, resulting in a clear definition of the microwire responses. The amplitudes in all three signal processing methods were distinguishable and sufficiently separated from the background (as shown in Figure 15). In such cases, the signal could be evaluated not only through ADC but also through precise analog comparators. The results of the evaluation using ADC are shown in Table 4.

From the original signal, it is evident that the microwire response is sufficiently large and clearly defined. Moreover, the noise, compared to the amplitude of the microwire responses, is so small that even when using analog comparators, it does not affect the measurement results. 

In this experiment, applying the chosen method again depends on the requirements. If it is necessary to measure quickly and accurately, using an analog comparator in conjunction with an RC filter is required. If hardware limitations are set and only software signal processing is needed, the ideal solution is to use an FIR filter. It provides the fast and precise processing of microwire responses. The sample averaging method can be used if slower but more accurate data processing is sufficient. The main difference between using/not using an RC filter is in the processing of the single period, where it is evident that the RC filter removes noise, enabling measurements to be performed through analog comparators. Other methods remain the same regardless of using/not using an analog RC filter.

### 3.3. Experiment with Feromagnetic Core and External Magnetic Background

**Figure 16:**
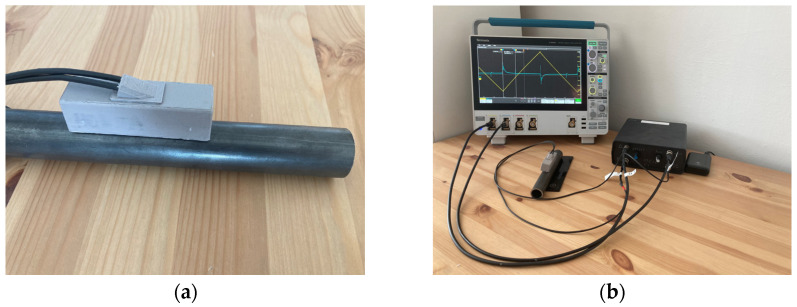
Experiment with a ferromagnetic core and external magnetic background. Figure (**a**) shows the coil system placed on the magnetic steel tube with a microwire inside. Part (**b**) shows the measurement under harsh conditions using analog RC filters. Part (**b**) also shows the measurement setup with the coil system, microwire (inside the magnetic steel tube), electronics, and Tektronix MSO44 oscilloscope.

In certain cases, measurements must be conducted under challenging, highly magnetic conditions. Any conditions (environment) introducing significant external magnetic fields into the measurement manifest on the sensing side with substantial noise and a heavily distorted signal background. Such conditions can be created, for instance, by magnetic steel near the measurement setup. The external magnetic field shifts the magnetization hysteresis loop of the microwire, which needs to be compensated for by a large excitation magnetic field to obtain a signal from the microwire. In this case, the signal background may be so distorted that comparators cannot be used for measurement, and therefore, it is necessary to work with the ADC sensing methods. The last part of the experiment shows a bistable microwire placed in a steel (magnetic) tube, with a coil system creating a strong excitation field from the ferromagnetic yoke. The measurement setup is shown in the following Figure 16.

#### 3.3.1. Experiment with Ferromagnetic Core, External Magnetic Background, and without Analog RC Filter

**Figure 17:**
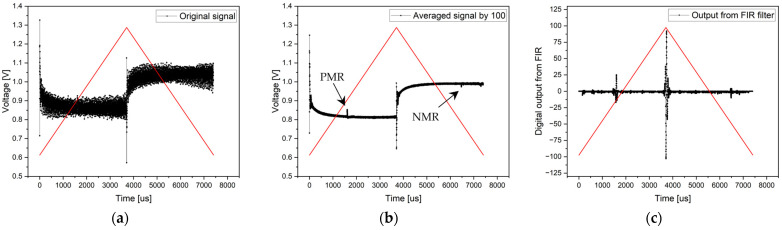
Results of the measurement with ferromagnetic core and external magnetic field. The electronics are designed without an analog RC filter. Graph (**a**) shows the original signal from the single-period processing method, chart (**b**) presents the results of the averaged signal from 100 periods, and chart (**c**) shows the output from FIR filtering.

The results of the last experiment found that the signal background was heavily distorted, containing additionally significant noise that completely masked the microwire responses. From the original signal obtained by the ADC of a single period, it was impossible to determine the positions of the microwire responses (see Figure 17—original signal).

The noise could be eliminated using additional digital signal processing methods. When averaging 100 consecutive periods, it was possible to observe the microwire responses (see Figure 17—averaged signal by 100). However, signal averaging failed to remove low-frequency background signals. The results of this method for the microwire response positions are shown in Table 5. 

Issues with background and noise are ideally addressed by FIR filtering. The background was completely smoothed out by applying an FIR filter, and high-frequency noise was removed as well (see Figure 17—output from the FIR filter). The microwire response positions were very easily detectable. The results are shown in Table 5. 

#### 3.3.2. Experiment with Ferromagnetic Core, External Magnetic Background and with Analog RC Filter

Good results were achieved once again by introducing an analog filter. The highest points of the microwire responses are several tens of millivolts above the background and noise. The microwire responses can be distinguished using all signal processing methods. 

**Figure 18:**
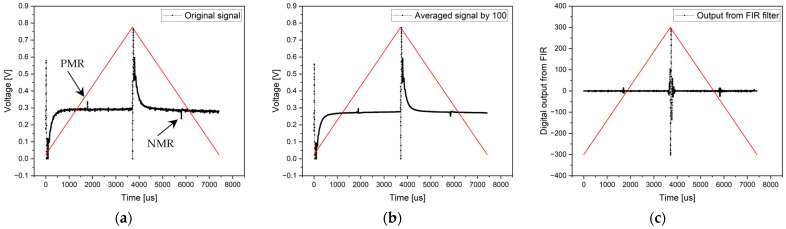
Results of the measurement with a ferromagnetic core and external magnetic field. The electronics contain an analog RC filter. Graph (**a**) shows the original signal from the single-period processing method, chart (**b**) presents the result of the averaged signal from 100 periods, and chart (**c**) shows the output from FIR filtering.

The method of sample averaging provided the same results as measuring the original signal. However, it significantly suppressed noise and created a larger difference between the background and the highest point of the microwire responses (see Figure 18). 

The last method, using FIR filters, eliminated all unnecessary harmonic components and clearly defined the positions of the microwire responses (with phase shift elimination) at the positions shown in Table 6.

**Figure 19:**
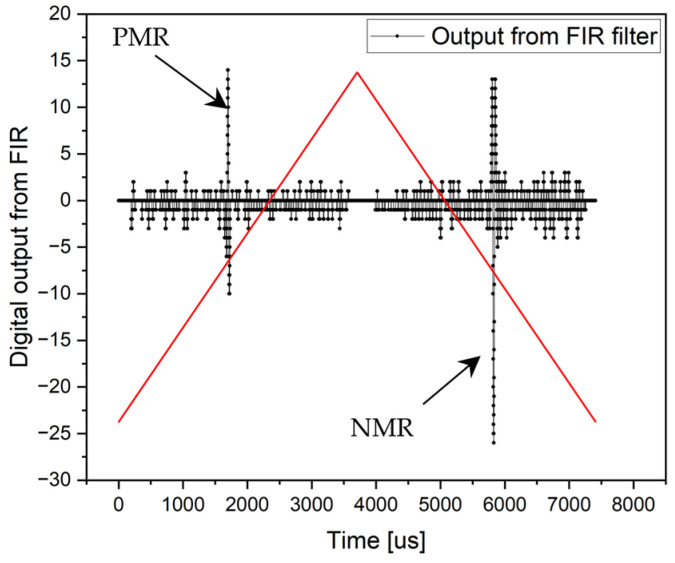
Output from the FIR filter where the DC value and changes in the slope of the triangular signal were removed from the searching area. In this case, both the microwire responses are clearly defined.

Although the microwire responses may appear small and difficult to distinguish, they are, conversely, very clearly defined during digital signal processing. The following figure demonstrates this. Although it may seem that the microwire responses are very small at the output of the FIR filter, the opposite is true. By removing (ignoring) the irrelevant areas of the period, which are the response of the pick-up coil to the change in the steepness of the triangular excitation signal (at the beginning and in the middle of the period), the microwire responses turn out to be clear and very distinct. This result is shown in Figure 19 (derived from Figure 18—output from FIR filter).

From the output, the signal is clearly visible, making it very easy for the microcontroller to find the maximum and minimum samples representing the microwire responses. The times at which these samples were recorded represent the switching time.

The outcome of the last experiment clearly defines the methods that cannot be applied. Signal processing without an analog RC filter is not feasible using single-period processing methods or analog comparator usage due to the signal background. Even the sample averaging method is hindered by the signal background. The only viable solution (without analog RC filters) is the application of FIR filters, which can accurately determine microwire responses. While applying an analog RC filter improves the situation, microwire responses are still too small, making it difficult to use analog comparators. Therefore, signal processing via FIR filters remains the fastest method, which does not require an analog RC filter or single-period processing with an analog RC filter. Accuracy and fluctuation removal can be achieved through sample averaging, provided that an RC filter is added to the electronic circuit, albeit at the expense of increased hardware demands.

## 4. Discussion 

The experiments mentioned above aim to investigate the possibilities of applying various digital signal processing methods using bistable microwires. Measurements were conducted in three experiments to assess the advantages and disadvantages of each method. In the first experiment, none, or a negligible magnetic background was present in the measurement device. The second experiment involved measurements with a ferromagnetic core, introducing a magnetic background that complicated signal processing. The final experiment measured a strong magnetic background from magnetic steel, significantly affecting the measurements. The task of the experiments was not only to measure the positions of the microwire responses and demonstrate the applicability of microwires under different conditions but also to determine the most suitable signal processing method in terms of speed and measurement accuracy. Based on these factors, it was then possible to determine the most suitable signal processing method, knowing where and under what conditions the measurements would take place.

According to the measurements, it was found that the fastest signal processing method for a 135 Hz signal was the application of comparators together with a 170 MHz timer. These could process the signal from the microwire response within one signal period. Thus, the results were evaluated instantly in real-time, corresponding to 7.407 ms. The second fastest signal processing method was the evaluation of single-period processing (the original signal), which took an average of 12 ms across all experiments. It indicates that processing requires one period for sampling and storing data in memory and a portion of the next period for evaluating the results. Another signal processing method was the application of an FIR filter, which evaluated results from one period. This method’s total processing and evaluation time of microwire responses was 50 ms. The slowest method involved averaging samples from 100 consecutive periods, which took nearly 750 ms.

On the other hand, the last-mentioned method (the sample averaging method) achieved the most accurate results among those utilizing ADC samples. Averaging samples stabilized the fluctuations of the microwire responses, resulting in precise results with a deviation of ±4µs. Although applying FIR filtering yielded less precise results, it was applicable in all experiments. Obtaining the results from one period is fast, but it may pose a risk of the microwire response fluctuations, displaying a deviation of up to 20 µs. Similarly, the single-period processing method, which evaluates the microwire responses from one period, operates with the same deviation. The result is evaluated from the current position of the microwire response, which may fluctuate slightly. Although this method is the fastest among those utilizing ADC and samples, it is difficult to apply to noisy signals. It should be noted that the sampling time of the ADC is 1.2354 µs, which presents an 810 kHz sample frequency. The method with the highest resolution is processing the microwire responses through comparators and a timer. The timer resolution is under 5.88 ns [40], allowing them to react quickly and precisely to the recorded microwire response. The results of this method are slightly shifted compared to those of methods utilizing ADC as they do not record the highest point but rather the intersection of the comparator’s reference value, which occurs before the microwire response reaches its peak. Nevertheless, the sensitivity of this method is incomparably higher than that of methods utilizing internal ADC, with measurements being 210 times more precise. This result is based on the frequency of the timer connected to the comparator and the sample rate of the AD converter (170 MHz/810 kHz). A significant disadvantage is that the method of using internal analog comparators cannot be applied to noisy signals.

Considering all the experiments described in the experimental part, it has been determined that the application of the described methods primarily depends on the usage requirements and the environment. If the application of a miniature coil system is sufficient and the signal is not influenced by a magnetic background, the fastest and most accurate measurement method is the application of analog comparators. In the case of this experiment, it was found that the use of analog RC filters is not necessary to achieve the same results (with/without RC filters).

The situation changed in the second experiment, where a ferromagnetic core was implemented into the coil system. It introduced magnetic background and noise into the signal, making it impossible to measure using analog comparators without an RC filter. A fast and accurate measurement with an analog comparator is possible, but only with hardware RC filtering. Suppose the situation limits hardware capabilities, where an RC filter cannot be used. In that case, the ideal solution is the application of FIR filters, which provide a fast and precise evaluation of microwire responses. However, a disadvantage is the phase shift of the output signal, which can be easily compensated. The method of sample averaging provides precise but time-consuming measurements.

However, the sample averaging method without an RC filter is impossible to use in harsh conditions, which was the subject of the third experiment with an external magnetic background. This experiment confirmed that measurements without an analog RC filter were only possible with the FIR filtering method. Applying other methods (without an RC filter) may have been difficult. Applying an RC filter also did not bring absolute clarity to the measurement in the case of comparator application due to the low amplitude of microwire responses. The sample averaging method and FIR filtering were applicable and produced good results.

## 5. Conclusions

This manuscript’s main topic was introducing, describing, and evaluating bistable microwires’ advantages in digital signal processing. The first advantage of bistable microwires is their bistable magnetization states. When the microwire magnetization changes, these states are presented as the microwire response on the sensing coil, the moment at which can be very easily measured over time. The second advantage is detecting and separating physical contributions such as external magnetic field, temperature, and mechanical stress. It means that simultaneously, it is possible to measure temperature/mechanical stress and the external magnetic field. Although this statement was not verified in this article because it was not our goal, according to [4,37], it is considered a general fact and property for bistable microcircuits. The last advantage of bistable microwires is the signal periodicity, meaning that the signal repeats periodically. This property allows for the utilization of digital signal processing. Thanks to this, it is possible to distinguish the signal of the microwire from the background and noise even in difficult conditions. Digital signal processing is precisely the main domain of signal processors, in this case, STM32G474RE microcontroller, thanks to which the measurement via bistable microwires is simple and achievable.

Overall, four signal processing methods from the microwire were used:

- An internal analog comparator processing with a timer of STM32G474RE.
- Single-period processing via internal ADC.
- Sample averaging via internal ADC.
- The utilization of a digital FIR filter.

Each signal processing method has its strengths and weaknesses. Therefore, to identify these advantages/disadvantages of each method, three experiments were conducted, allowing for the determination of optimal solutions. Each experiment also included testing with/without the application of an analog RC filter:

1. An experiment without a magnetic background.
2. An experiment with a magnetic background in the excitation coil.
3. An experiment with a ferromagnetic core and external magnetic field.

From the experiments and applied methods, it was found that each method has its precise application in specific areas. The choice of which method of processing microwire responses to use depends primarily on the application requirements and environment. It can be determined whether very fast measurements are required or slower measurements with higher accuracy are needed. On the other hand, it is necessary to consider the environment and whether the method can be applied due to possible noise. It has also been found that the ideal approach is to combine some methods. For example, FIR filtering is applicable in all experiments and is 15 times faster than averaging 100 periods. Therefore, it is possible to utilize a combination of filtering from multiple periods, achieving high accuracy in a relatively short time. The FIR filtering method is accurate, fast, and applicable in all cases. However, it is not more precise and faster than the signal processing method using analog comparators, which is the fastest method with the highest resolution. This method is limited by the amount of noise that can be filtered by an analog RC (if the situation allows). Suppose it is necessary to display the entire period of the signal. In that case, it is suitable to use ADC methods, with single-period processing being the fastest evaluation method if the situation allows it. The sample averaging method is more precise but time-consuming. Therefore, the result is a clear combination of individual methods with or without the use of an analog RC filter. Applying all the methods in practice is based on the requirements of the specific situation and environment.

## Figures and Tables

**Table 1:** Microwire response positioning and switching times of the experiment without a magnetic background or analog RC filter.

Signal Processing Method	Positive Microwire Response	Negative Microwire Response	*t*_1_ (µs)	*t*_2_ (µs)	Processing Time (ms)
Reference positions	-	-	2002	6443	-
Original signal	1617	5220	1998	6449	12.177
Averaged signal by 100	1620	5214	2001	6441	748.107
Output from FIR filter	1619	5203	2000	6428	50.219
Analog comparators	340,789	1,093,830	2004	6434	7.407

**Table 2:** Microwire responses position and switching times of the experiment without magnetic background and with analog RC filter.

Signal Processing Method	Positive Microwire Response	Negative Microwire Response	*t*_1_ (µs)	*t*_2_ (µs)	Processing Time (ms)
Reference positions	-	-	2304	5995	-
Original signal	1863	4845	2302	5985	12.864
Averaged signal by 100	1867	4856	2306	5999	748.107
Output from FIR filter	1860	4844	2297	5984	50.219
Analog comparators	388,960	1,008,440	2288	5932	7.407

**Table 3:** Microwire responses position and switching times of the experiment with magnetic background in excitation coil and without analog RC filter. The analog comparator was not applicable in this case (N/A).

Signal Processing Method	Positive Microwire Response	Negative Microwire Response	*t*_1_ (µs)	*t_2_* (µs)	Processing Time (ms)
Reference positions	-	-	1875	6312	-
Original signal	1519	5086	1876	6283	12.571
Averaged signal by 100	1518	5113	1875	6316	748.107
Output from FIR filter	1516	5089	1872	6287	50.219
Analog comparators	N/A	N/A	N/A	N/A	N/A

**Table 4:** Microwire responses position and switching times of the experiment with magnetic background in excitation coil and with analog RC filter.

Signal Processing Method	Positive Microwire Response	Negative Microwire Response	*t*_1_ (µs)	*t*_2_ (µs)	Processing Time (ms)
Reference positions	-	-	1810	6409	-
Original signal	1475	5195	1822	6418	11.973
Averaged signal by 100	1466	5189	1811	6410	748.107
Output from FIR filter	1460	5184	1800	6404	50.219
Analog comparators	303,324	1,085,889	1784	6387	7.407

**Table 5:** Microwire responses position and switching times of the experiment with ferromagnetic core, external magnetic background and without analog RC filter. The analog comparator was not applicable in this case (N/A).

Signal Processing Method	Positive Microwire Response	Negative Microwire Response	*t*_1_ (µs)	*t*_2_ (µs)	Processing Time (ms)
Reference positions	-	-	1275	6475	-
Original signal	-	-	-	-	-
Averaged signal by 100	1340	5241	1277	6474	748.107
Output from FIR filter	1320	5248	1274	6483	50.219
Analog comparators	N/A	N/A	N/A	N/A	N/A

**Table 6:** Microwire responses position and switching times of the experiment with ferromagnetic core, external magnetic background and with analog RC filter. The analog comparator was not applicable in this case (N/A).

Signal Processing Method	Positive Microwire Response	Negative Microwire Response	*t*_1_ (µs)	*t*_2_ (µs)	Processing Time (ms)
Reference positions	-	-	1900	5845	-
Original signal	1573	4682	1943	5784	12.169
Averaged signal by 100	1537	4735	1899	5849	748.107
Output from FIR filter	1542	4720	1904	5831	49.437
Analog comparators	N/A	N/A	N/A	N/A	N/A

## Data Availability

The data presented in this study are available on request from the corresponding author. The data are not publicly available due to the continuation of this study and the fact that the data are still being worked on.
